# Focusing on Long COVID and HIV Prevention

**DOI:** 10.31486/toj.25.5056

**Published:** 2025

**Authors:** Ronald G. Amedee

**Affiliations:** Director of Clinical School and Professor, The University of Queensland Medical School, Ochsner Clinical School, New Orleans, LA; Editor-in-Chief, *Ochsner Journal*

*Autumn is the antidote to stifling summer.*

–*Terri Guillemets*

The Fall 2025 issue of *Ochsner Journal* contains two editorials, three original research articles, one quality improvement article, and six case reports and clinical observations. The first editorial, “When We Don’t Have All the Answers: Long COVID and the Need for Humility in Medicine,” was submitted by Ochsner senior physician Liza Di Leo Thomas who recounts her difficult battle with post-acute sequelae of SARS-CoV-2 infection and urges physicians to listen with humility and compassion to their patients who present with inexplicable symptoms. Traditionally underrecognized, infection-associated chronic conditions are real, and patients have dealt with their life-altering effects for years, often without being believed by their physicians. This situation is changing, however, because the increasing incidence of Long COVID worldwide has led the medical establishment to take a close look at these debilitating conditions.

The second editorial, “‘Not Today’: Overcoming PrEP Hesitancy, Stigma, and Adherence Barriers With Lenacapavir,” submitted by Margaret Conrad and Meredith Clement, physicians at Emory and LSU, discusses the potential for lenacapavir, the recently FDA-approved HIV prevention option, to dramatically reduce the risk of HIV infection. In clinical trials of this twice-yearly injection, nearly 100% of HIV infections were averted in 2 large clinical trials.

The first original research article in the fall issue, “Maternal Quality of Life Following a Periviable Delivery,” is from a team at the University of Alabama at Birmingham and explores the psychological and social health of mothers who delivered preterm infants. The second original research article details “Using Machine Learning to Identify Geographic and Socioeconomic Disparities in Dialysis Facility Outcomes Across the United States” and was authored by Ashkar from Ochsner Lafayette General and Gottumukkala from the University of Louisiana at Lafayette. Completing this section is “Accuracy of Conversion Disorder Diagnosis via Telestroke Network Consultation: A Retrospective Cohort Study” that has several authors from the Ochsner Neuroscience Institute: Poongkunran, Vidal, Iwuchukwu, McGrade, Chehebar, and Zweifler.

The quality improvement submission, “Low Unintended Dural Puncture Rate Using a Flush-Measure-Check-Advance Technique to Perform Combined Spinal-Epidural Anesthesia in Parturients: A Quality Improvement Clinical Series,” comes from primary authors affiliated with LSU anesthesiology, physicians Riopelle and Gayle, in collaboration with Ochsner research statistician Jeff Burton.

The case reports and clinical observations include “Hydrocodone-Induced Delirium in an Otherwise Healthy 20-Year-Old Male” by Ochsner Health emergency medicine physicians Ginsberg and Chauhan. Teams from Lankenau Medical Center in Wynnewood, Pennsylvania, contributed two cases to the issue: “A Rare Cause of Internal Jugular Vein Thrombosis: Deep Tissue Massage” and “Rare Diagnosis of Localized Breast Amyloidosis in the Setting of Lymphoplasmacytic Lymphoma.” Both short-term and long-term complications of weight-loss surgery are explored in “Concurrent Wernicke Encephalopathy and Posterior Reversible Encephalopathy Syndrome Following Gastric Sleeve Surgery,” authored by clinicians at the University of Pittsburgh and Duke University medical centers, and in “Ischemic Necrosis of the Gastric Remnant 42 Years After Bariatric Surgery” from authors affiliated with Rocky Vista University Colorado. “Mixed Neuroendocrine Non-Neuroendocrine Neoplasm of the Ampulla of Vater: Report of a Rare Location,” was authored by four surgeons, a pathologist, and a radiologist at the All India Institute of Medical Sciences in Jodhpur, India.

This edition of the *Journal* will be published near the middle of September 2025. Our days at this time of year in New Orleans often still bring daily high temperatures in the low 90s, but on rare occasions, we awaken to lows in the 70s with much reduced humidity, making us aware that the season (and time) are about to change. The other notable change that has recently occurred but will continue through early 2026 is the return of American football. Whether you follow high school, collegiate, and/or NFL teams, this time of year is a great season to head out and view a game under the lights as you also enjoy a slight chill in the evening air. Happy fall to all!

